# Neural Responses in Parietal and Occipital Areas in Response to Visual Events Are Modulated by Prior Multisensory Stimuli

**DOI:** 10.1371/journal.pone.0084331

**Published:** 2013-12-31

**Authors:** Hamish Innes-Brown, Ayla Barutchu, David P. Crewther

**Affiliations:** 1 The Bionics Institute, Melbourne, Australia; 2 Swinburne University of Technology, Melbourne, Australia; 3 Deakin University, Melbourne, Australia; 4 Florey Institute of Neuroscience and Mental Health. Melbourne, Australia; 5 Centre for Human Psychopharmacology, Swinburne University of Technology, Melbourne, Australia; Centre de Neuroscience Cognitive, France

## Abstract

The effect of multi-modal vs uni-modal prior stimuli on the subsequent processing of a simple flash stimulus was studied in the context of the audio-visual ‘flash-beep’ illusion, in which the number of flashes a person sees is influenced by accompanying beep stimuli. EEG recordings were made while combinations of simple visual and audio-visual stimuli were presented. The experiments found that the electric field strength related to a flash stimulus was stronger when it was preceded by a multi-modal flash/beep stimulus, compared to when it was preceded by another uni-modal flash stimulus. This difference was found to be significant in two distinct timeframes – an early timeframe, from 130–160 ms, and a late timeframe, from 300–320 ms. Source localisation analysis found that the increased activity in the early interval was localised to an area centred on the inferior and superior parietal lobes, whereas the later increase was associated with stronger activity in an area centred on primary and secondary visual cortex, in the occipital lobe. The results suggest that processing of a visual stimulus can be affected by the presence of an immediately prior multisensory event. Relatively long-lasting interactions generated by the initial auditory and visual stimuli altered the processing of a subsequent visual stimulus.

## Introduction

When a brief flash stimulus is accompanied by two brief sounds, the single flash is sometimes perceived as two discrete flashes. This phenomenon has been termed the ‘fission’ illusion [1]. The illusion has since been shown to be robust to temporal delays of up to approximately 100 ms between the auditory and visual stimuli [2], spatial separation of the auditory and visual stimuli across the visual midline [3], and even accuracy feedback on each trial specifically designed to reduce its strength [4].

Several neuro-imaging studies [5]–[8] have shown that the perception of the fission illusion is correlated with increased activity in the primary visual cortex. These studies generally support the hypothesis that the illusion results from the integration of auditory and visual information, rather than the possible introduction of response biases. Similarly, two flashes presented with a single sound can ‘fuse’ into a single flash percept. Neural correlates of the fusion illusion, measured using functional magnetic resonance imaging (fMRI) [9], [10] and event-related potentials (ERPs) [6], are correspondingly reduced in the same areas. More recently, a trans-cranial direct current stimulation (tDCS) study showed that perception of illusory flashes increases with anodal (excitatory) stimulation of temporal areas, and decreases when occipital areas are stimulated [11]. Disruption of the right angular gyrus in parietal cortex by trans-cranial magnetic stimulation (TMS) also reduces susceptibility to the illusion [12]. Thus, the perception of illusory flashes may be dependent on cortical temporal-occipital interactions.

Together, the findings reviewed above that describe increased activity in primary visual areas during perception of illusory extra flashes (fission) and reduced activity in the same areas during illusory reduction of flashes (fusion) strongly suggest that the illusion occurs as a result of modulation of activity in primary visual cortex by neural processes related to the auditory stimulus. That the illusion can be enhanced or degraded by stimulation or disruption of parietal and temporal areas also suggests that these higher-order regions have a possible role to play, although the mechanism by which this might occur is not currently understood. The flash-beep illusions obviously do not occur when a single beep is presented alone. As Meylan & Murray [13] have suggested, the context (the preceding stimuli) thus has a role to play in the generation of the illusion.

In this study, we presented fission and fusion illusion stimuli as well as a number of visual-only and congruent audio-visual control stimuli which were very similar to the illusion stimuli but where illusions did not occur. EEG was recorded during the presentation of the stimuli to examine the brain’s responses to the second flash stimulus depending on whether it was preceded by a uni-sensory or multi-sensory stimulus.

## Methods

### Ethics statement

The protocol was approved by the Swinburne University Human Experimentation Ethics Committee, and written, informed consent was obtained from all participants.

### Participants

Eleven participants were recruited from the student body at the Brain Sciences Institute at Swinburne University. Age ranged from 22–32 years (*M* = 28.6, *SD* = 3.5), and four were male.

### Stimuli and apparatus

The stimuli and apparatus were the same as those used in Innes-Brown and Crewther [3], except that the refresh rate of the monitor was increased to 75 Hz, and the timing of the auditory and visual stimuli were slightly adjusted such that they occurred simultaneously, rather than with the 23ms delay used previously. The delay between auditory and visual stimuli is not critical to the perception of the illusion. Shams et al [2] found that illusion reports remained strong when the beep stimulus was presented within 115 ms either side of the flash stimulus, with maximum illusion strength at approximately 0 ms (no delay). This stimulus timing of 0 ms (no delay) is simpler to implement and interpret, especially in the context of ERP analysis, and has been used successfully in fMRI [8] and ERP [13] investigations of the flash-beep illusion.

**Figure 1:**
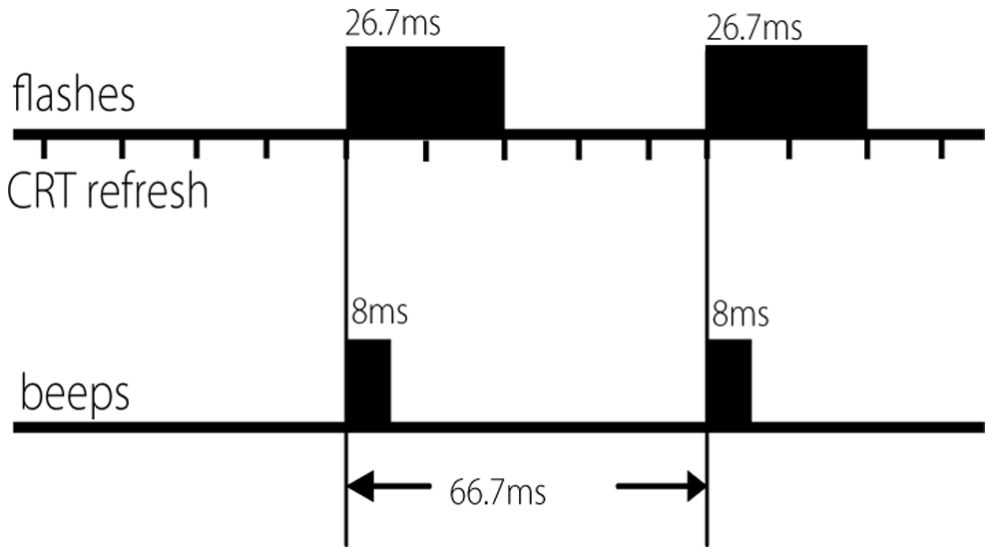
Flash beep stimulus timing. Shown is an example of a 2-flash, 2-beep (2F2B) trial.

The experiment was conducted in a quiet, sound-treated, and electrically shielded room. The background sound level was approximately 39 dB SPL (A-weighted). The visual stimulus consisted of a white disk, which flashed once or twice at full brightness and approximately 100% contrast on a 15-inch cathode ray tube monitor (CRT: Philips 107E) with a black background, in a darkened room. The disk subtended 3° of visual angle and was located 7.5° below a fixation cross, which was positioned 2.5° above the centre of the screen. Each flash consisted of two refresh periods (26.67 ms, see Figure 1). On selected trials, short beeps were presented simultaneously with the flashes, from a speaker placed centrally under the CRT. The beep was a 3500 Hz, 83 dB SPL (A-weighted) sine wave of 8 ms duration, with 3 ms rise and fall times. Participants sat in a comfortable chair with their head 100 cm from the CRT. A keyboard rested on a desk at a comfortable distance directly in front of the CRT. The auditory and visual stimuli were controlled using Presentation 10.1 (Neurobehavioural Systems). Using a cathode-ray oscilloscope, photo-diode and microphone, the average delay between the visual flash stimulus and the first measurable peak of the auditory stimulus was.3 ms, with no measureable standard deviation for 20 repetitions.

### Procedure

Multi-modal fission and fusion illusion stimuli were presented, along with uni-modal visual control stimuli (a single or double flash), and congruent multi-modal stimuli, where the number of flashes and beeps was equal. In each trial, there was either a single or double flash, along with zero, one, or two beeps. Trial types will be henceforth referred to using a code indicating the number of flashes followed by the number of beeps – ‘2F2B’ thus refers to a trial with two flashes and two beeps. The six possible trials types were therefore 1F0B, 1F1B, 1F2B (fission illusion), 2F0B, 2F1B (fusion illusion) and 2F2B. In trials with multiple flashes or beeps, the time between the onsets of successive beeps or flashes was 66.7 ms, corresponding to 5 CRT refresh periods. There was always a flash at 0 ms – therefore if the number of flashes and beeps was not equal (1F2B and 2F1B), there was always a combined flash/beep, followed by either a single flash or a single beep. An example of a 2F2B trial is shown in Figure 1.

The fixation cross was displayed alone for an interval that varied randomly in each trial between 1200 and 1500 ms. This random variation was introduced in order to reduce the possibility of participants predicting the stimulus onset and responding too quickly, and to reduce the possibility of readiness or contingent negative variation potentials occurring in the pre-stimulus EEG [14], [15]. The flash/beep sequence then began. Following the sequence was another short randomly varied interval (1200 to 1500 ms), after which the text “How many flashes did you see?” was displayed in place of the fixation cross. This text remained in place until the participant made a response on the keyboard, or until 2.5 seconds passed, after which time the response was deemed invalid. Participants were instructed to keep their gaze on the fixation cross during each trial and count the number of flashes that would appear whilst ignoring the beeping sounds. The response was made after each trial by pressing keys labelled ‘1’ or ‘2’ on a keyboard.

The six possible flash/beep stimuli were presented in pseudo-random order 20 times each in a single block. This block was repeated 5 times (with trials re-randomised each time). Each stimulus was thus presented 100 times. Keyboard responses were recorded for each stimulus presentation. Each block ran for an average duration of 10 minutes and breaks could be taken between each block. The total testing time was approximately 1.5 hours, including breaks and instruction time.

### Electrophysiological recordings

The continuous EEG was recorded from 60 sintered silver/silver-chloride electrodes mounted in an elastic cap according to the international 10–20 system. The continuous EEG was amplified, filtered with a bandpass of 0.1–100 Hz, and digitised at a sampling rate of 1000 Hz using a Synamps II EEG amplifier.

## Analysis and Results

### Behavioural data

Accuracy scores were analysed in order to firstly determine whether participants could accurately count the visual flash stimuli either in the absence of an auditory stimulus or with a congruent number of beeps, and secondly to determine the extent of fission and fusion illusions reported in trials where an illusion was expected. SPSS version 17 was used for all statistical analyses.

For each of the six stimulus types, accuracy scores were calculated by dividing the number of correct responses by the total number of responses made, so that non-responses (when the participant took longer than 2.5 seconds to respond) were not counted as incorrect. Non-responses were rare (.9%). For the non-illusion trials (1F0B, 1F1B, 2F0B, 2F2B), the accuracy scores reflected the degree to which participants were able to accurately count the visual flash stimuli with no beeps or with a congruent number of beeps. Conversely, for the illusion trials, low accuracy scores indicated the presence of illusory perception. In fission trials (1F2B), low accuracy indicated that *more* flashes were reported than were presented, and in fusion trials (2F1B), low accuracy indicated that *less* flashes were reported than were presented.

**Figure 2:**
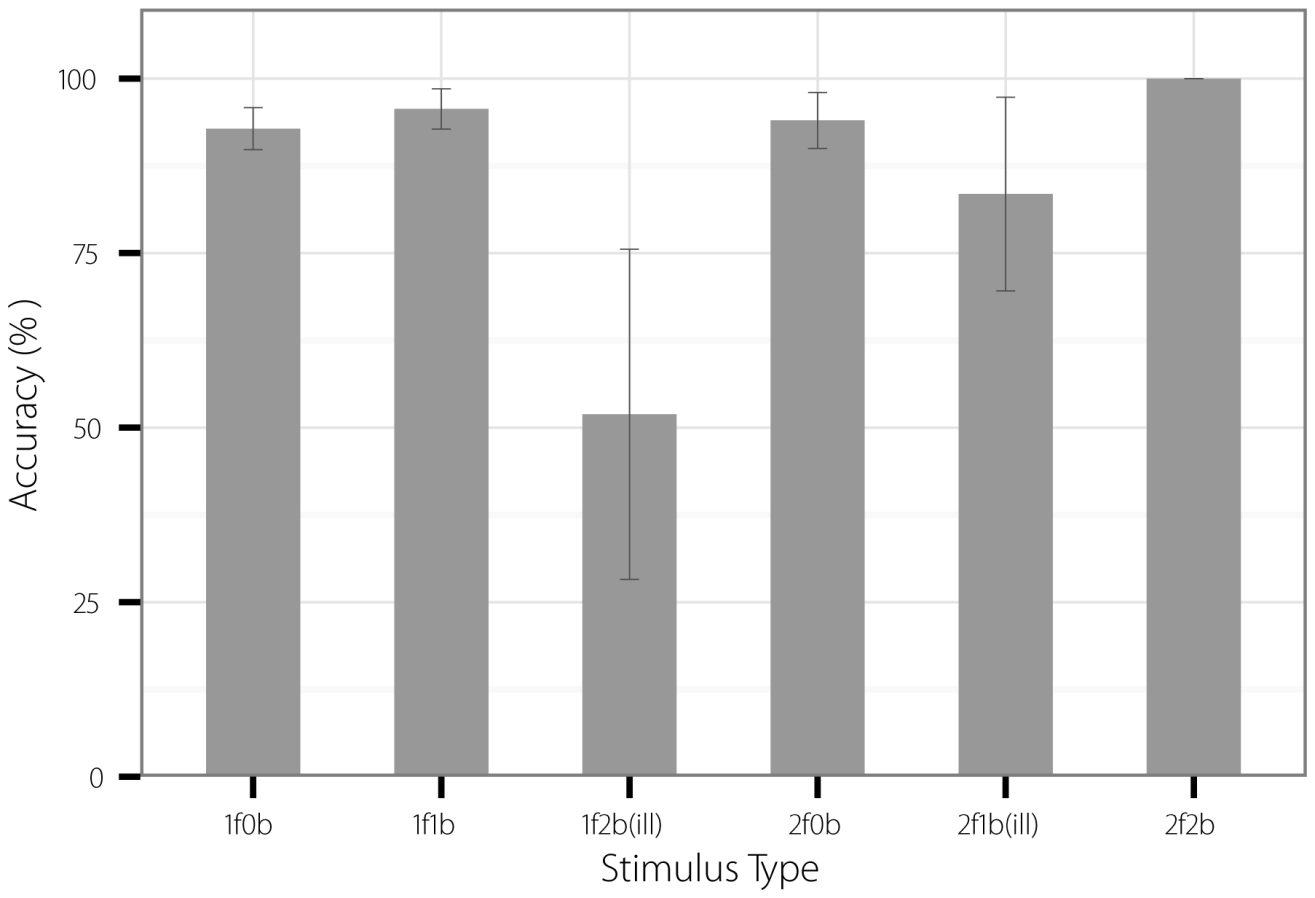
Mean accuracy for counting flashes (± SEM) for each stimulus type. In fission illusion trials (1F2B), participants often reported more flashes than were present; in fusion illusion trials (2F1B), participants sometimes reported less flashes than were presented.

Figure 2 shows mean accuracy scores for each stimulus type. In general, all participants responded with a high level of accuracy for all non-illusion stimuli, suggesting that the visual stimuli were not ambiguous and that the visual flashes could be counted relatively easily. This was the case both in the visual-only uni-modal trials (1F0B and 2F0B) as well as in multi-modal congruent trials where the number of auditory and visual stimuli were equal (1F1B and 2F2B). However, accuracy was lower and variability higher for both types of illusion trials (fission – 1F2B, and fusion – 2F1B).

The significance of these effects was assessed using a repeated measures analysis of variance (ANOVA) with within-subjects factors for Nflash (1 flash, 2 flashes), and Nbeep (0 beeps, 1 beep, 2 beeps). There were significant main effects of Nflash, *F*(1,10)  = 10.8, *p* = .008, *η* ^2^ = 52, and Nbeep, *F*(2,20)  = 8.3, *p* = .002, *η* ^2^ = .45, as well as a significant Nflash x Nbeep interaction, *F*(2,20)  = 17.7, *p*<.001, *η* ^2^ = .64. Accuracy in the illusion trials compared to the corresponding non-illusion trials was examined by decomposing the interaction using simple effects analysis [16]. Pairwise comparisons with Sidak adjusted alpha levels are reported throughout. In one-flash trials, accuracy scores were lower in fission illusion trials compared with both the uni-modal (1F0B), *p* = .003, and congruent multi-modal control trials (1F1B), *p* = .002. In two-flash trials, accuracy in the illusion trials (2F1B) was lower than in the congruent multi-modal trials (2F2B), *p* = .02, but not compared to the uni-modal trials (2F0B), *p* = .06.

As the number of males (N = 4) and females (N = 7) were unequal, 2-sample t-tests were performed on accuracy scores for each stimulus type, in order to test for any bias in accuracy based on gender. Accuracy scores were not significantly different between males and females for any stimulus.

Thus, both the flash-beep fission and fusion illusions were present, although the fusion illusion was weak, and performance in the 2F1B condition (where the fusion illusion might be expected to occur) was over 80% on average.

### EEG data

**Summary of methods.** The analysis partly followed the methods described in Meylan and Murray [13]. In this approach, only four stimulus types were analysed – uni-modal stimuli, which were the 1F0B and 2F0B stimuli, and multi-modal stimuli, which were the 1F1B and 2F1B stimuli. The response to the second flash only was isolated by defining UNI and MULTI difference waves as follows: UNI  =  2F0B – 1F0B, and MULTI  =  2F1B – 1F1B. In this way, the effect of either a uni-modal or multi-modal preceding context on the response to the second flash could be determined. For subtractions involving the 2F1B stimulus (which could potentially evoke a ‘fusion’ llusion), only correct (non-illusory) responses were analysed.

Both the UNI and MULTI difference waves represent the neural response to only the second flash. The comparison of the UNI and MULTI difference waves was therefore designed to determine the possible timing and location of statistically significant differences between the UNI and MULTI difference waves. Rather than focus on the identification and measurement of ERP components (such as the N1, P2, etc), the analysis sought firstly to determine time points at which statistically significant differences between the UNI and MULTI difference waves occurred, and secondly to use source localisation methods to locate the parts of the brain in which these differences were likely to have occurred.

The global field power (GFP) was also calculated. GFP is a measure of the overall electrical field response at the scalp for each time point. It has the advantage that it is not affected by the choice of reference electrode, and the corresponding disadvantage that it provides no information about the possible neural sources underlying the electric field measured at the scalp. Hence in the current study, it was used to determine time periods at which the overall electrical response to the UNI and MULTI stimuli differed from each other, without requiring any prior assumptions of electrode location, reference configuration, or neural source location. The sources underlying the electric response in these time periods were then estimated using source localisation techniques constrained to the relevant time periods.

**EEG pre-processing procedures.** The continuous EEG was filtered using a second-order bi-directional (zero-phase-shift) Buttterworth bandpass filter with cutoff frequencies of.1–45 Hz and slopes of 12dB/oct. Following filtering, the EEG was re-sampled at 500 Hz, and epochs from –200 to 800 ms post-stimulus were constructed. Once segmented, artefact rejection and correction procedures were applied. A three-step approach was taken: 1) EEG epochs and channels containing artefacts were rejected. Only artefacts that were considered to be severe, or of a unique ‘stereotyped’ nature likely to cause an unsuccessful independent components analysis (ICA) decomposition were rejected [17] as implemented in EEGLAB [18]. 2) ICA was run, and components representing ocular or other artefacts were marked and removed using the ‘ADJUST’ plugin [19]. 3) The independent component time-courses were re-projected back into EEG epochs, and previously deleted channels re-interpolated. Finally, the average of all trials (the ERP) for each electrode, and the standard deviation across electrodes (equivalent to the mean global field power – GFP [20]) was calculated for each participant and stimulus-response combination.

***When*** **- determining time periods when statistically significant differences occurred.** Although the final determination of time intervals was calculated using the GFP, the scalp ERP data for the four stimuli, as well as the UNI and MULTI differences waves, were also visualized to allow comparison with previous work.

**Figure 3:**
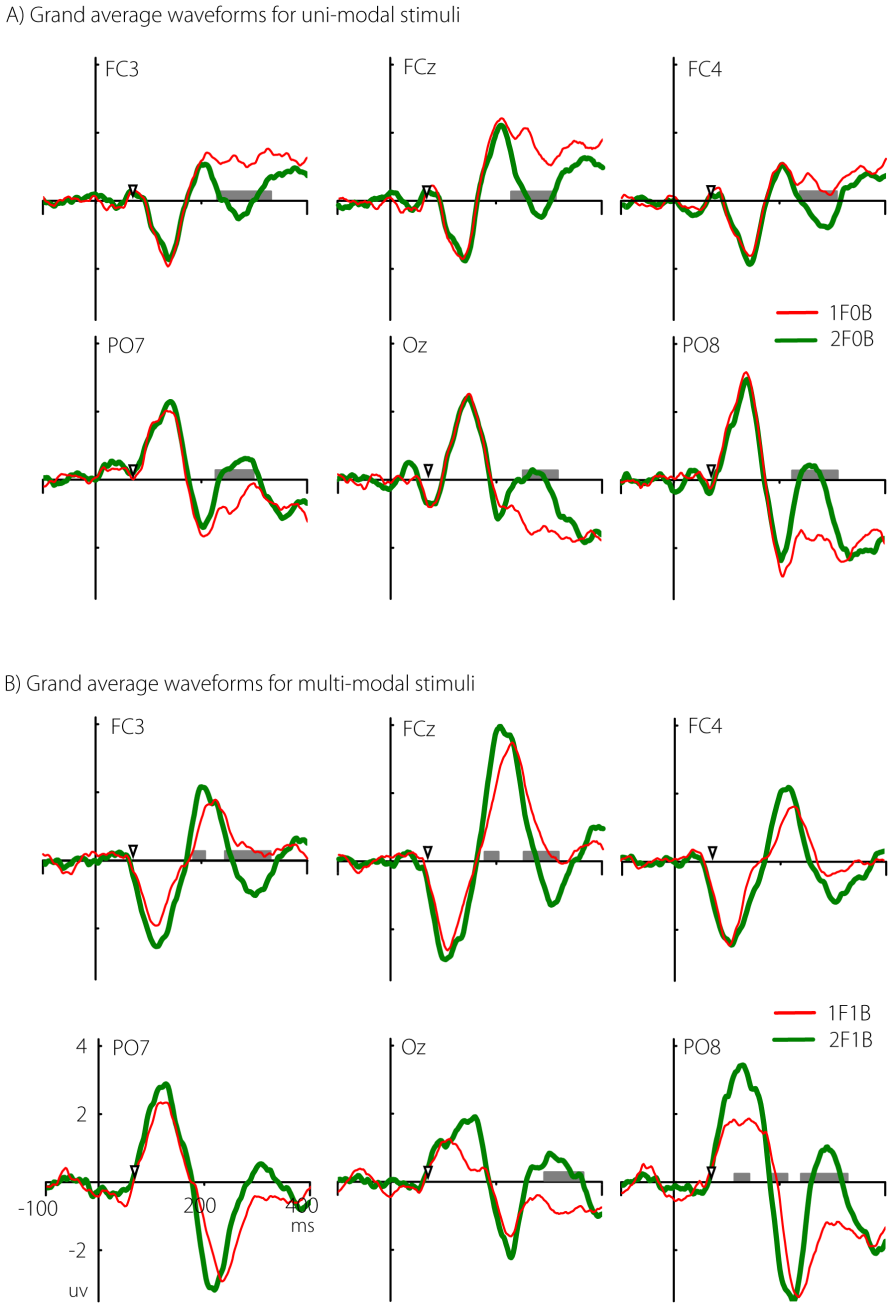
Grand average ERPs for UNI (top panel) and MULTI (bottom panel) stimuli. Significant differences between the waveforms are marked with grey boxes (permutation test, p <.05). The x-axis represents time relative to the first flash. The time of the second flash (67 ms) is marked on the x-axis.

The analysis focussed first on visualising differences in the timing, magnitude, and topography of ERPs in response to the uni-modal and multi-modal stimuli used to calculate the UNI and MULTI difference waves. The statistical significance of differences between the ERPs in each condition were visualised by using point-wise non-parametric multiple permutation tests with 2000 permutations (as implemented in the EEGLAB functions ‘std_stat’ and ‘statcond’ [21]), for each time point and electrode. Throughout, similar permutation tests were used to test for statistically significant differences between pairs of waveforms. This method allowed the visual identification of periods of statistically significant differences between waveforms in a manner more conservative than standard parametric t-tests, as no assumptions of normality were required. In addition, only periods of significant differences longer than 10 samples (20 ms) were considered reliable[22]. This approach was used for visualisation purposes only, and has been used in similar studies previously [23]–[25], but could be under-conservative as the procedure was applied without an exact estimate of the first-order autocorrelation and with the whole epoch length rather than a small segment as the original technique was intended to be used. We emphasize that these analyses are intended only to provide visualisation of the effects within the data, and our main analysis was based principally on the reference-independent GFP data, where additional measures were taken to control for the false-discovery rate inherent in mass-univariate testing.Figure 3 shows the grand average ERPs at six electrode sites, calculated for the uni-modal (1F0B and 2F0B) and multi-modal (1F1B and 2F1B) stimuli. The periods of time where the two waveforms significantly differed are highlighted with grey boxes. Visual inspection, combined with the exploratory permutation tests, showed that the grand average waveforms for the uni-modal stimuli (1F0B and 2F0B) show the expected visual evoked potential (VEP) morphology, with strong N1 and P1 peaks visible, especially at the occipital electrodes (Figure 3). Note that these data are displayed with the average reference, rather than the nose reference often used in VEP studies. Also visible are differences between the 1F0B (light/red) and 2F0B (heavy/green) ERPs. Differences likely reflecting the response to the second flash at 66.7 ms occurred in central, parieto-occiptal and fronto-central sites from around 220-300 ms post-stimulus.

ERPs in response to the multi-modal stimuli (1F1B and 2F1B) showed a similar overall morphology to the uni-modal stimuli, although overall amplitudes were larger, probably reflecting the summation of auditory and visual ERPs (Figure 3). There were again differences between the waveforms at similar latencies to the uni-modal stimuli, as well as at earlier latencies. The spatial pattern of significantly different intervals appeared more broadly spread in time than with the UNI stimuli, extending both earlier and later. It should be noted that no attempt was made to control for multiple comparisons among electrodes in these statistical displays. The statistical display is intended only to aid visual inspection.

**Figure 4:**
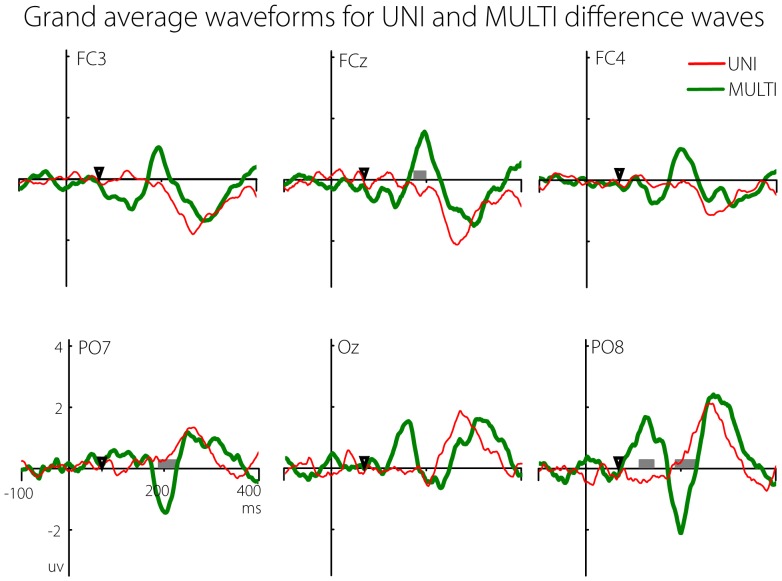
Grand averages for UNI (red) and MULTI (green) difference waves. Grey boxes show time points at which a permutation test indicated a significant difference between the waveforms. The x-axis represents time relative to the first flash. The time of the second flash (67 ms) is marked on the x-axis.

**Comparison of ‘UNI’ and ‘MULTI’ difference waves.** In order to compare responses to the second flash as a function of the preceding uni-modal or multi-modal stimulus, the UNI and MULTI difference waveforms were calculated. Figure 4 shows the UNI and MULTI difference waveforms at six electrodes, averaged across all participants. Recall that both waveforms represent the isolated response to the second flash, with the only difference being the immediately prior stimulus. The UNI waveform shows the response to the second flash when it was preceded by another flash stimulus, while the MULTI waveform shows the response to the second flash when it was preceded by a multi-modal flash/beep stimulus. Any differences between these two waves were thus due to the differential effect of the preceding stimulus. Among the six electrodes shown in Figure 4, differences were present at around 200 ms at fronto-central sites, whereas earlier differences were present at parieto-occipital sites. There was a complex pattern of early and late differences across the entire electrode array.

**Figure 5:**
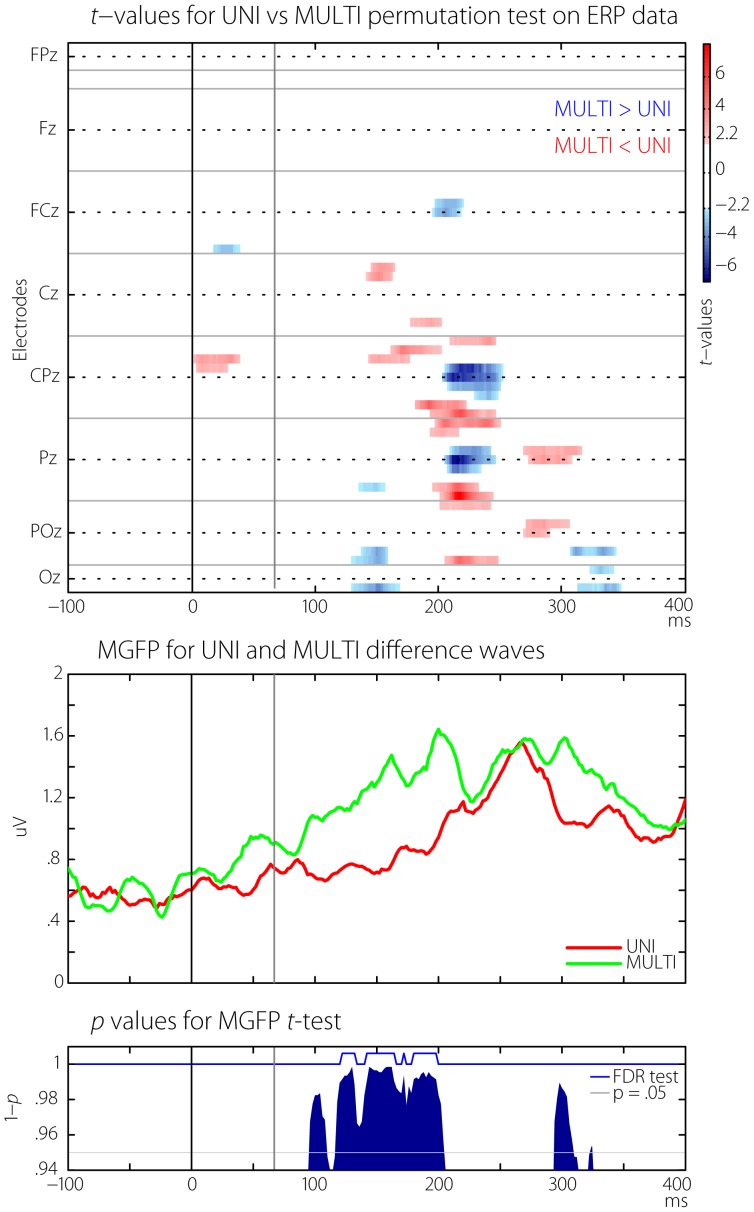
Results from statistical testing of UNI vs MULTI difference waves. The x-axis on all plots represents time relative to the first flash. The grey line at 67 ms indicates the time of the second flash. TOP: t-values from point-wise permutation testing between the UNI and MULTI difference waves at every electrode. Electrodes are arranged in bands separated by grey horizontal lines from the frontal (top of plot) to occipital (bottom of plot) regions. Within each band, electrodes are arranged from left-most (top of band) to right-most (bottom of band). The midline electrode in each band is shown on the y-axis. For example, in the lowest (occipital) band, the three electrodes shown are O1, Oz and O2. MIDDLE: The mean (across participants) global field power (MGFP) calculated across all electrodes for UNI and MULTI difference waves. The MULTI waves shows increased activity in early and late time windows. BOTTOM: p-values from a point-wise permutation test between the two GFP waveforms show that the MULTI difference waveform is larger for a very short period at around 50 ms, and extended periods from 100–200 ms, and from 300–350 ms. Time points where false-discovery rate thresholding of the p-values was significant are indicated with the blue line.

In order to simplify the display of the UNI and MULTI ERP data across all electrodes, the *t*-statistic from a point-wise permutation *t*-test between the UNI and MULTI waves for each electrode was plotted on a common axis. Figure 5 (top panel) shows the results. Only *t*-values larger than the critical *t*-value for *df*  =  10 and extending over at least 10 consecutive time points (20 ms) are shown. This display is essentially a repeat of the data shown in Figure 4, but all electrodes are shown on a common x-axis, and critical *t*-values testing the difference between the waveforms are shown rather the separate voltages for each waveform.

As can be seen, there were three main intervals over which differences occurred. The first was a very early difference from 0–40 ms at central and lateral sites. These differences were before the presentation of the second flash (at 67 ms) and were not analysed further. From 120–250 ms there followed a large number of differences across many electrodes. At frontal sites the UNI waveform was mostly larger than the MULTI waveform (red clusters), whilst the opposite was the case at more parietal electrodes (blue clusters). There were also later differences, particularly a cluster of occipital sites where the MULTI response was larger than the UNI response.

However, it should be emphasized that both the ERP voltage plots and *t*-statistic intensity plot only provided information regarding the time periods and scalp electrodes at which differences were found using the particular reference configuration in this study (the average reference), and are not generalizable.

In order to determine appropriate time periods in which to localize the cortical sources of the differences, the reference-free GFP measure was used. The GFP was determined for each participant by calculating the standard deviation across all electrodes at each time point. This is equivalent to the sum of squares of potential differences at all possible electrode combinations, and indicates the overall strength of the electric field at the scalp at each time point. In order to more strictly determine the time intervals over which significant differences between the GFP related to the UNI and MULTI difference waves occurred, the false-discovery rate method (FDR [26], as implemented in the ‘FDR.m’ EEGLAB function [18]) was used to control for the possibility of Type I errors caused by the large number of permutation tests used.

The grand average GFP for the UNI (red line) and MULTI (green line) difference waves is shown in the middle panel of Figure 5. The bottom panel shows the *p*-values (only those with greater than 10 consecutive significant tests) from the point-wise permutation test between the two GFP waveforms. In addition, the black line shows when the FDR-corrected p-values are significant. There are two features of note. First, it is clear that the MULTI difference wave showed larger overall response strength than the UNI difference wave. Secondly, the differences were significant using the Guthrie & Bachwald [22] criteria in two main intervals – an early interval from 120–190 ms, and a later interval from 300–320 ms. However, when the stricter FDR criterion was applied, the differences were only significant in the first interval. Thus, the overall electric field measured at the scalp in response to the second flash was significantly larger in two specific intervals when it was preceded by a multi-modal flash/beep stimulus compared to when it was preceded by a flash alone, although only the first interval survived the stricter FDR-based correction for multiple comparisons. Both intervals were submitted to the subsequent source analysis (detailed below), but results from the second interval must be interpreted with caution.

***Where*** **- source analysis of statistically significantly different time periods.** In order to determine the most probable locations of cortical generators underlying the differences found in electrical field strength measured at the scalp, Standardised Low-Resolution Electromagnetic Tomography (sLORETA) [27], [28] was used to estimate the cortically-constrained current source density of the UNI and MULTI difference waves for each participant.

**Figure 6:**
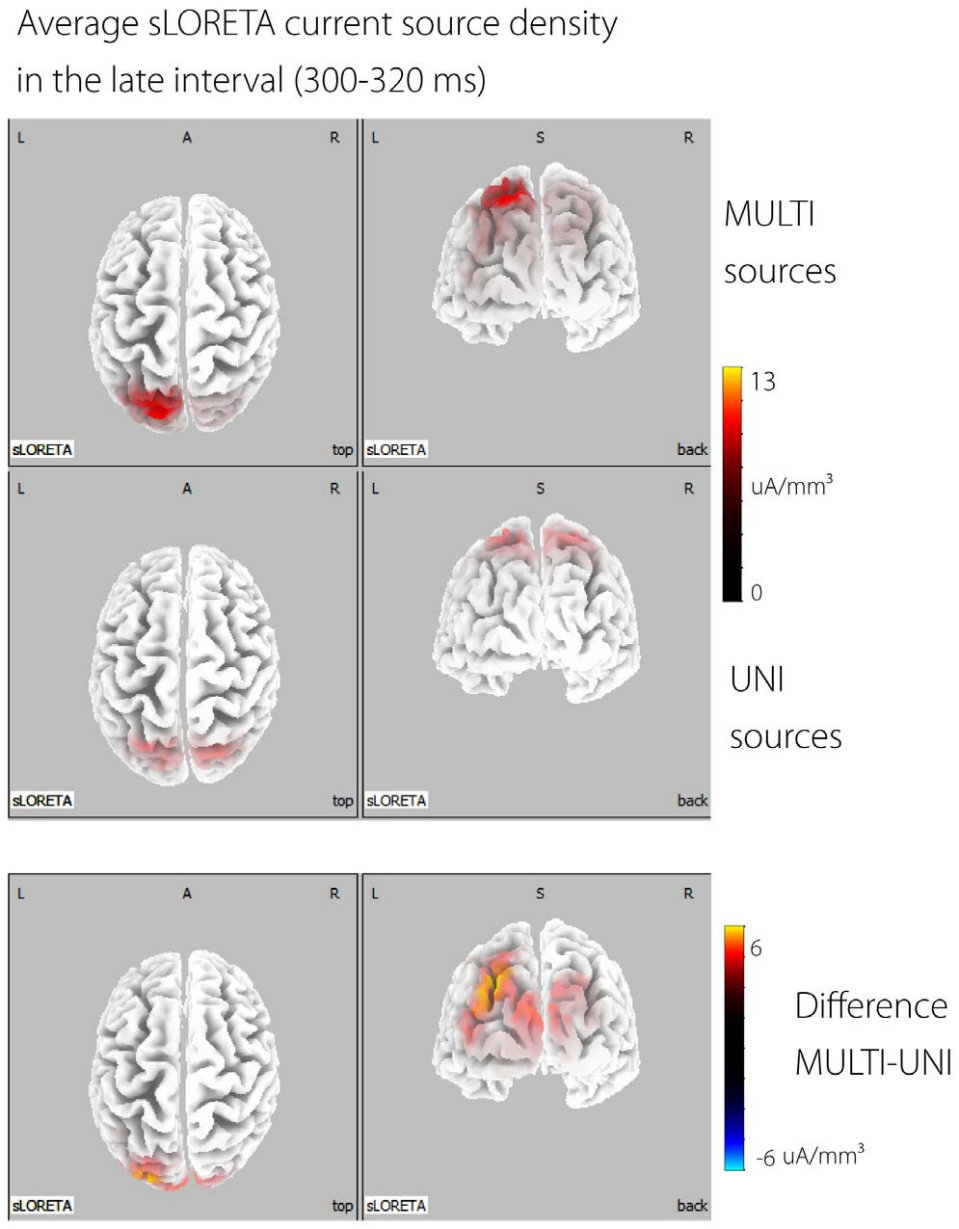
sLORETA source localisation. Current source density (µA/mm3) displayed on the MNI Colin-27 T2 template brain for the MULTI (top row) and UNI (bottom row) waves, in the late interval (300–320 ms). The difference between the two (MULTI-UNI) is shown in the bottom row. Four views are shown: from left to right these are top, back, left, and right views.

sLORETA solutions for all time points and all participants were first calculated for the UNI and MULTI waves. Average sLORETA images were calculated for the UNI and MULTI waves for each participant in the two time segments that were revealed as significant by the GFP permutation test. Figure 6 shows sLORETA CSD maps plotted on the MNI template brain [29] for the UNI and MULTI responses in the late interval (only the late interval is displayed for brevity), as well as the difference between the two (MULTI - UNI). The main sources for both were in the parietal and occipital lobes, and the CSD values for the MULTI responses were generally higher than for UNI responses. The differences were mainly focussed in the occipital lobes.

**Figure 7:**
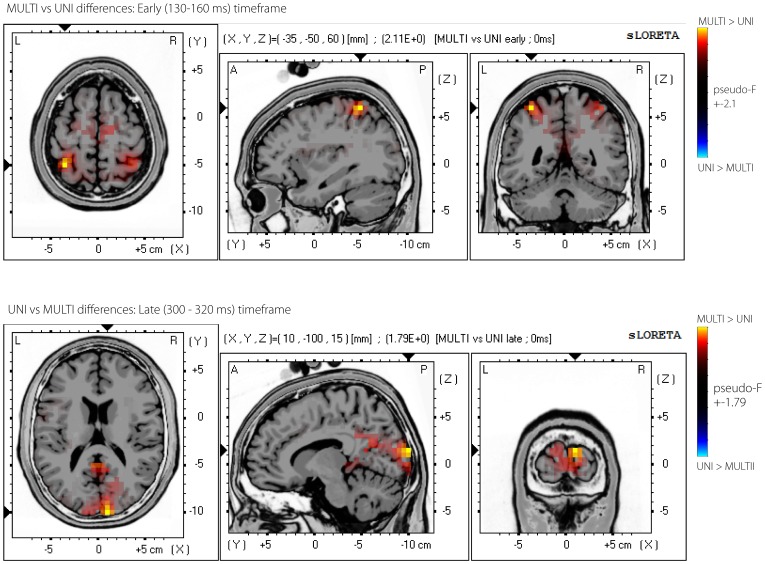
sLORETA source localisation. sLORETA statistical image showing significant pseudo-F values from a voxel-wise random permutation test between MULTI and UNI sLORETA source localisations in the early (130–160 ms) and late (300–320 ms) timeframes. The pseudo F-statistics for voxels with significant differences were plotted in their appropriate locations in Talairach space on the MNI Colin-27 T2 template brain.

To determine the statistical significance of differences between localisations for the UNI and MULTI difference waves, ‘Statistical non-Parametric Mapping’ (SnPM) was used, as implemented in the sLORETA software [27]. SnPM performed voxel-wise randomisation tests (5000 permutations), and calculated critical thresholds and *p*-values corrected for the number of comparisons involved in the voxel-wise test. The log ratio of averages (similar to the *F*-statistic) was calculated for every voxel, and thresholded with alpha level of.05 (see Holmes et al [30] and Nichols et al [31]). The pseudo *F*-statistics for voxels with significant differences were plotted in their appropriate locations in Talairach space on the MNI ‘Colin27’ T2 template brain [29]. Figure 7 shows these statistical difference maps for the early (top row) and late (bottom row) time intervals. The MNI co-ordinates of the location of the maximum or minimum pseudo-*F* statistic was converted to a brain region using the Talairach map [32], and are listed in Table 1. It should be noted that neither individual MRI images nor exact electrode positions were available and so were not used to calculate the EEG sources. Using sLORETA and a similar template brain model and standard electrode positions, Valdez-Hernandez et al [33] found mean locatisation errors of approximately 6.4 mm.

**Table 1:** Locations of the maximum difference (maximum pseudo-*F* statistic) in the early and late time intervals.

Timeframe	Location of maximum difference	Location of secondary maximum difference	Pseudo-*F* for UNI ≠ MULTI	*p*
Early 130–160 ms	(–35, –50, –60 mm), BA 5: post-central gyrus, superior parietal lobe.	BA 40: Inferior parietal lobule, parietal lobe	2.11	.001
Late 300–320 ms	(10, –100, 15mm), BA 18: cuneus, occipital lobe	BA 17: cuneus, occipital lobe	1.80	.02

In the early timeframe (130–160 ms post-stimulus), sources in the post-central gyrus of the superior parietal lobe (Brodmann area 5) were significantly more active for the MULTI difference wave compared to the UNI difference wave (Figure 7, top row). The second-largest difference was found in the inferior parietal lobule (Brodmann area 40).

In the late timeframe (300–320 ms post-stimulus), sources in the occipital lobes (Brodmann areas 18 and 17 had the highest and second-highest pseudo-*F* values, respectively) were significantly more active for the MULTI difference wave compared to the UNI difference wave (Figure 7, bottom row).

## Discussion

The experiment found that the electric field strength related to a flash stimulus was stronger when it was preceded by a multi-modal flash/beep stimulus, compared to when it was preceded by another uni-modal flash stimulus. This difference was found in two distinct timeframes – an early timeframe, from 130–160 ms, and a late timeframe, from 300–320 ms. The differences in the later time interval did not survive strict controls over the false-discovery rate due to the large number of multiple comparisons and hence should be interpreted with caution. However, source localisation analysis found that the increased activity in the early interval was localised to an area centred on the inferior and superior parietal lobes, whereas the later increase was associated with stronger activity in an area centred on primary and secondary visual cortex, in the occipital lobe. The results suggest that processing of a visual stimulus can be affected by the presence of an immediately prior multisensory event. Relatively long-lasting interactions generated by the initial auditory and visual stimuli altered the processing of a subsequent visual stimulus.

### Involvement of the parietal lobe

In the early interval (130–160 ms), increased activity in inferior and superior parietal lobes was found. The parietal lobes have traditionally been considered ‘association cortex,’ where information from separate sensory processing pathways is combined to form a unified sensory space [34]. Imaging studies in humans have found the area to be both multi-modal, or responsive to stimulation in more than one modality, as well as an area of integration, displaying non-linear super- or sub-additive response characteristics when more than one sensory mode is activated [35], [36].

Direct connections between the parietal and auditory cortex and the visual cortex have been found using tracer techniques in primates [37]–[39]. Reciprocal connections from visual cortex to caudal auditory areas have also been found more recently [40]. Intra-cranial recordings in awake humans undergoing surgical planning procedures for intractable epilepsy have provided a timeline of the visual, auditory, and auditory-visual activity in these regions [41]. In that study, a detection task using simple auditory, visual, and audio-visual stimuli was employed. Although no illusory stimuli were presented, the short combined flash/beep stimulus was very similar to the multi-modal context stimulus used in the current study. The accuracy and reaction times to the audio-visual stimulus in Molholm et al [41] indicated that the facilitation of behavioural responses was not simply due to the summation of probabilities of responses for the two uni-modal stimuli alone. Grid electrodes over the parietal cortex showed onsets of neural responses to the auditory stimuli at around 30 ms, and onsets to the visual stimuli at around 75 ms. Non-linear responses to the audio-visual stimuli (where the response to the audio-visual stimuli was significantly different than the sum of responses to the auditory and visual stimulus alone) were found in the same locations as those activated with the uni-modal stimuli with onsets from between 120–160 ms.

Together with the neuroimaging data [35], [36], the study by Molhom et al [41] suggests that the parietal lobes are sites of multisensory *integration*, and not only co-activation. The results also provide a timeline of activation in the parietal lobes by auditory, visual, and audio-visual stimuli. Interestingly, increased MULTI responses in the early interval from the current study were also found in the parietal lobes, at a very similar time to that found by Molholm et al [41]. The initial flash/beep multi-modal context stimulus in the current study was very similar to the multi-modal stimulus used in Molholm et al [41], suggesting the possibility that the increase in activity found in the current study was also indicative of multi-sensory processes in the parietal lobes, driven by the combined audio-visual context stimulus.

### Timing of interactions found in other flash-beep studies

**Figure 8:**
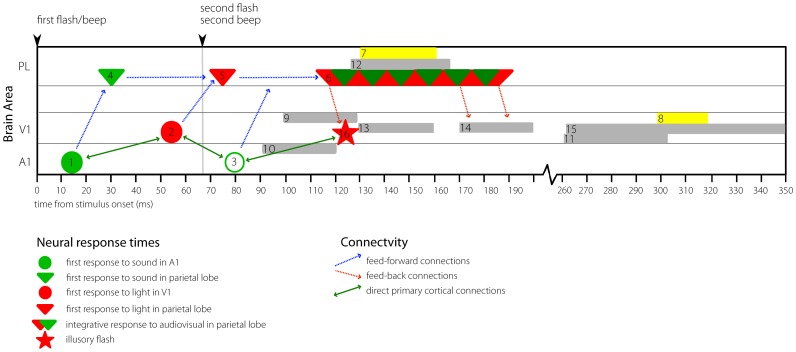
Timing Diagram. Diagram showing various responses to a simultaneous flash/beep stimulus at time zero. A1 – Primary Auditory Cortex, V1 – Primary Visual Cortex, PL – Parietal Lobes. See text for explanation of numbered points.

Figure 8 shows a timeline positioning the results of the current study (yellow boxes) alongside reported timing of the first neural responses in A1, V1, and the parietal lobes (PL), as well as multi-sensory interactions found from various flash-beep illusion studies to combined flash/beep stimuli (grey boxes). Figure 8 is complex and each part will be discussed in turn. Before discussion of the illusion studies, an overview of the networks engaged by the presentation of a single simultaneous flash/beep stimulus is in order.

The simplest case is when a single beep is paired with a flash. This was the stimulus configuration in Molholm et al [41]. The stimulus used in that study is very similar to the multi-modal context stimulus in the current study, and this combined flash/beep stimulus was also present for each of the illusion-capable stimuli in the current study as well as others - 1F2B and 2F1B stimuli both contain a combined flash/beep stimulus at 0 ms. In this simplest case, the green and red dots on the diagram (Figure 8, labels 1 and 2) show the initial activation of auditory and visual primary sensory cortex, respectively. As can be seen, the first activation of auditory cortex is considerably faster, around 10–14 ms [42], than the first activation of visual cortex, around 50 ms [43]. Although the studies of first neural responses in primary auditory and visual areas were performed using multi-unit activity studies in primates, the timing for this discussion has been extrapolated to human-equivalent times using the ‘3/5ths rule’ [44], [45]. Rapidly following each of these primary sensory activations, there is activation of the parietal lobe (green and red inverted triangles) [41], firstly by the auditory stimulus, at about 30 ms (Figure 8, label 4), and then by the visual stimulus at around 70 ms (Figure 8, label 5). Feed-forward connections from primary sensory areas to parietal areas are shown with dotted blue arrows [46]. As the auditory and visual stimuli were presented together, there is also a non-linear interaction (Figure 8, label 6) in the parietal lobe, beginning from around 120–160 ms [41]. Subsequent feed-back connections from the parietal lobes back to primary sensory areas are shown with dotted red arrows [37]. The parietal lobes are known to have top-down influences on multisensory processes in other cortical areas, such as the primary auditory and visual areas, as well as in subcortical areas such as the superior colliculus [47]. Direct cortico-cortical connections between A1 and V1 are shown with solid brown arrows [38], [39]. In summary, the presentation of a simultaneous flash/beep stimulus activates the auditory cortex, the visual cortex, and the parietal cortex (and other regions not discussed here). Multi-sensory interactions occur in the parietal cortex beginning from approximately 120–160 ms after the presentation of the multi-modal stimulus. Feedback connections may be active from parietal areas to primary sensory areas, as well as between primary sensory areas.

Although ERP responses to the fission illusion stimuli were not tested in this study, the fission illusion stimulus (1F2B, or a combined flash/beep followed by another beep) has an onset with the same characteristics (a combined flash/beep) as the multi-modal context stimulus in the current study, and similar characteristics to the multi-modal stimulus in Molholm et al [41]. As single beeps presented in isolation are not known to elicit flash illusions, the effect of the multi-modal stimulus preceding the second *beep* in the fission illusion stimulus is likely to be involved in triggering the second beep to elicit an illusory flash sensation, in the same way that the multi-modal stimuli in the present study affected subsequent processing of the second flash. The findings suggest multiple mechanisms by which the illusion may operate: these include direct cortico-cortical connections between A1 and V1, feedback connections from the parietal cortex, or by some combination of the two. There may also be networks involving cortico-thalamic loops [48], [49], but these possibilities are difficult to address with the EEG measures used in the current study. Figure 8 shows hypothetical activation of the auditory cortex by the second *beep* in the 1F2B illusion stimulus (Figure 8 label 3, open green circle at 80 ms) and the approximate time at which the illusory flash might be expected to occur (Figure 8, red star, label 16). As the timing of the perception of the illusory flash has not been experimentally determined, this position is speculative, and is based on the timing of activation reaching V1 from a second ‘real flash,’ if one were to occur simultaneously with the second beep. Given this caveat, however, it is interesting to compare this proposed timing of the illusory flash with the multi-sensory interactions found in the parietal lobe elicited by the initial flash/beep stimulus (Figure 8 label 6) [41], as well as the results from the current study, where increased activation of the parietal lobes was found in isolated responses to the *second flash* at the same time (Figure 8, point 7). The fact that ERP responses to the second flash in our 2F1B stimuli were modulated by the presence of the initial multi-modal context stimulus, in the same timeframe and in the same brain areas where multi-sensory interactions to the *initial* flash/beep context stimulus were found by Molholm et al (2006), suggests that processing of the second flash was affected by continuing multi-sensory processes in the parietal lobes that were most likely triggered by the initial multi-modal context stimulus.

### Illusions and involvement of the occipital lobe

The results reviewed above describe the effect of a single multi-modal stimulus on subsequent uni-sensory processing. By comparing the responses to 1F2B stimuli to the sum of 1F0B and 0F2B responses, a number of flash-beep illusion studies have also found non-linear interactions related to the perception of the illusory second flash in the same ∼110–160 ms timeframe and in similar brain regions. This interaction waveform is commonly referred to in the literature, and is usually written as AV– (A + V). Selected results from these studies are depicted using grey rectangles in Figure 8. For instance, the MEG study of Shams et al [50] found illusion stimulus interactions at both parietal *and* occipital MEG sensors in the same 120–160 ms timeframe as the multi-sensory interactions from Molholm et al [41] and the current study found in the parietal lobes only (Figure 8, labels 12 and 13). Mishra et al [6] also found interactions in the same timeframe: an increase of ERP activity localised to V1 just prior to the expected time of the illusory flash was found by these authors, but only in a subset of participants who were pre-disposed to the illusion (Figure 8, label 9). Prior to the increased activity in V1, an enhanced negativity localised to A1 was also found, this time in trials where the illusion actually occurred compared to those where it did not occur (Figure 8, label 10). While activation of V1 (30–60 ms after the second beep) was necessary but not sufficient for perceiving the illusory flash, the earlier negativity in A1 (only 20–40 ms after the second beep) in illusion trials only appeared to be the obligatory trigger for the illusion. These authors proposed that rapid interplay at least partly via direct cortico-cortical connections between A1 and V1 was responsible for perception of the illusory flash.

Shams et al [7] investigated the effect of auditory stimuli on the flash visual-evoked potential (VEP), using a slightly altered version of their original flash-beep paradigm. ERPs were examined by comparing the 1F2B responses to the sum of the flash alone (1F0B) and beeps alone (0F2B) stimuli. Using t-tests, the amplitude of each point in the average waveforms was then compared against zero. When the stimuli were presented in the visual periphery, an ‘early’ interaction was found at occipital electrodes between 170 and 200 ms, and a ‘late’ interaction from 260 to 360 ms (Figure 8, labels 14 & 15). The early time interval corresponded to ∼90–140 ms after the second beep – the stimulus that is presumably responsible for the generation of the illusion. The authors therefore interpreted this effect as an indication that the second beep ‘activated’ the primary visual cortex, thus generating the percept of the illusory second flash.

As well as the early differences in the parietal lobes, indications of stronger activity related to the second flash when it was preceded by a multi-modal context stimulus were also found in the current study in the occipital lobes at the relatively late time of 300–320 ms (Figure 8, label 8). The differences between the UNI and MULTI waveforms in this later time interval did not survive strict controls over the false-discovery rate due to the large number of multiple comparisons and hence should be interpreted cautiously. However, this time and location corresponds with the finding of ‘late’ interactions (1F2B > (1F0B + 0F2B)) at occipital electrodes in Shams et al [7] (compare with Figure 8 label 15), as well as the decreased activity in MULTI compared to UNI difference waves in Meylan and Murray [13] (compare with Fig 8 label 11). Shams et al [7] compared the 1F2B > (1F0B + 0F2B) interaction waveform with activity at the same electrode evoked by a real flash (ie 2F – 1F), and found that the waveforms were indistinguishable. They therefore concluded that the interaction waveform reflected modulation of visual-specific processing by the auditory stimulus. It is also interesting to compare the current results with those of Watkins et al [8], who found that fMRI activation in retinotopically-mapped V1 was stronger for 1F2B stimuli on trials when illusory flashes were reported compared to when the stimulus was perceived veridically. As has been previously mentioned, the 1F2B stimulus contains the same initial multi-modal flash/beep stimulus that was considered as the ‘context’ stimulus in the current study. In the current study, it was followed by another flash stimulus, and the activity related to another flash/beep stimulus, but without any following second flash, was then subtracted, leaving only the response to the second flash. To follow the analogy, in Watkins et al [8] the multi-modal flash/beep stimulus was followed by another *flash,* and it was found that activity in V1 increased when this extra flash also caused the perception of an additional beep. As with the current study, the modulation of visual processing that led to increased activity in V1 in Watkins et al [8] may have been caused either by direct cortico-cortical connections from A1 to V1, or by the modulation of V1 by multi-sensory processes set in motion by the initial multi-modal flash beep stimulus. These possibilities could be further explored in future research using Granger causality or transfer entropy measures.

Overall, incorporating the results from the current study with previous studies showing multisensory interactions in the parietal lobe following a combined flash/beep stimulus suggests an involvement of both feedback from parietal to primary sensory areas, as well as direct connections between primary sensory areas.

### Comparison to Meylan & Murray (2007)

The present study was similar in design to Meylan and Murray [13]. However, there are several important differences in the results. Firstly, there were small differences in the behavioural results – while Meylan and Murray [13] report very high accuracy rates (M = 89%) for the fusion (2F1B) stimulus, accuracy rates were slightly lower in the current study (M = 77.2%, SD = 29.1), indicating the presence of fusion illusions on a small number of trials in the current study. However in both studies, only correctly-responded trials were analysed. In other respects, the behavioural results were very similar, with accuracy above 90% for all non-illusion stimuli in both studies. For the fission (1F2B) stimulus, mean accuracy was close to 50% in both studies, a figure consistent with the majority of flash-beep illusion studies.

Meylan and Murray [13] found only one time interval in which the electric field response for the UNI and MULTI waves (also measured using the GFP) was different. This interval was from 238–275 ms, directly in-between the two significant intervals found in the present study. The other major difference lies in the direction of the result. Whereas Meylan and Murray found the MULTI GFP waveform to be of overall lower amplitude than the UNI GFP waveform, and significantly lower in the aforementioned time interval, the MULTI GFP waveform was consistently larger than the UNI waveform in the current study.

The differences between the two results may be due either to participant factors or to experimental methods/analysis factors. The age ranges were very similar between the two studies, although while the sample in the current study was predominantly female (7 females, 4 males), the sample in Meylan and Murray [13] was predominantly male (6 males, 2 females). However, accuracy scores for any stimulus in the current sample did not differ significantly for males and females, making the gender distribution an unlikely source of the difference in results. Similarly, there were small differences in the stimulus timing used. Table 2 shows stimulus details for both studies. While the duration of the visual stimulus were longer in the current study, the auditory stimulus was slightly shorter, and the onset times of the auditory and visual stimuli were identical. Differences in the stimulus durations are unlikely to account for the differences in the neurophysiological results, although it is possible that the longer flash duration and subsequent reduction in the blank inter-stimulus interval between flashes in the current study may have rendered the visual double-flash stimulus more likely to ‘fuse’ into a single flash, thus accounting for the difference in accuracy scores for the 2F1B trials. Participants in the current study were instructed to ‘count the number of flashes that they saw while ignoring the beeping sounds.’ The exact instructions are not reported in Meylan and Murray (2007), however the procedure section states that ‘Subjects’ task was to indicate the number of flashes perceived via a serial response box.’

**Table 2:** Stimulus configuration differences between the current study and Meylan & Murray [13].

Study	SOA	Refresh rate	Flash Duration (refresh periods)	Flash location	Flash size	Beep duration	N (males, females)
Current study	66.7 ms	75 Hz	26.7 Hz (2)	7.5° below fixation	3°	8 ms	11 (4,7)
Meylan & Murray 2007	66.7ms	75 Hz	13.3 Hz (1)	6.3° below fixation	2.2°	13 ms	8 (6,2)

Differences in the EEG recording setup and analysis are also unlikely to account for the differences in results. Although Meylan and Murray [13] used 128 recording sites rather than the 60 used in the current study, the increase in the number of electrodes is more likely to affect source localisation accuracy rather than the GFP measure (although it is acknowledged that the overall magnitude of the GFP will likely decrease as the number of electrodes increases due to the central limit theorem). In many other respects the analysis of EEG data was similar – the average reference was used, the UNI and MULTI subtractions were performed in the same way, and the GFP was calculated in the same way. In short, it is difficult to explain the large differences in result between the two studies. Individual differences and the composition of the samples in each case may explain the differences in results.

## Conclusion

Overall, the results from the current experiment showed that neural responses related to processing of a visual stimulus were modulated by prior multisensory stimuli. Specifically, EEG responses to a flash stimulus were stronger when a flash stimulus was immediately preceded by a multi-modal flash-beep stimulus compared to when it was preceded by another uni-modal flash. The differences were localised to the superior and inferior parietal cortex from 130–160 ms, and to the primary and secondary visual cortex from 300–320 ms. The results are supportive of views implicating the involvement of higher-order multi-sensory association regions in uni-sensory processing, but cannot rule out the involvement of direct cortico-cortical connections between primary sensory areas.
